# Semaphorin7A promotes tumor growth and exerts a pro-angiogenic effect in macrophages of mammary tumor-bearing mice

**DOI:** 10.3389/fphys.2014.00017

**Published:** 2014-02-05

**Authors:** Ramon Garcia-Areas, Stephania Libreros, Samantha Amat, Patricia Keating, Roberto Carrio, Phillip Robinson, Clifford Blieden, Vijaya Iragavarapu-Charyulu

**Affiliations:** ^1^Tumor Immunology, Department of Biomedical Sciences, Florida Atlantic UniversityBoca Raton, FL, USA; ^2^Immunology, Department of Biological Sciences, Florida Atlantic UniversityBoca Raton, FL, USA; ^3^Tumor Immunology, Microbiology and Immunology, University of Miami Miller School of MedicineMiami, FL, USA; ^4^Department of Clinical Sciences, Florida Atlantic UniversityBoca Raton, FL, USA; ^5^Department of Pathology and Laboratory Medicine, Jackson Memorial Hospital, University of Miami Miller School of MedicineMiami, FL, USA

**Keywords:** SEMA7A, angiogenesis, macrophages, breast cancer, CXCL2/MIP-2, MMP-9

## Abstract

Semaphorins are a large family of molecules involved in axonal guidance during the development of the nervous system and have been recently shown to have both angiogenic and anti-angiogenic properties. Specifically, semaphorin 7A (SEMA7A) has been reported to have a chemotactic activity in neurogenesis and to be an immune modulator through α1β1integrins. SEMA7A has been shown to promote monocyte chemotaxis and induce them to produce proinflammatory mediators. In this study we explored the role of SEMA7A in a murine model of breast cancer. We show that SEMA7A is highly expressed by DA-3 murine mammary tumor cells in comparison to normal mammary cells (EpH4), and that peritoneal elicited macrophages from mammary tumor-bearing mice also express SEMA7A at higher levels compared to those derived from normal mice. We also show that murine macrophages treated with recombinant murine SEMA7A significantly increased their expression of proangiogenic molecule CXCL2/MIP-2. Gene silencing of SEMA7A in peritoneal elicited macrophages from DA-3 tumor-bearing mice resulted in decreased CXCL2/MIP-2 expression. Mice implanted with SEMA7A silenced tumor cells showed decreased angiogenesis in the tumors compared to the wild type tumors. Furthermore, peritoneal elicited macrophages from mice bearing SEMA7A-silenced tumors produce significantly (*p* < 0.01) lower levels of angiogenic proteins, such as CXCL2/MIP-2, CXCL1, and MMP-9, compared to those from control DA-3 mammary tumors. We postulate that SEMA7A in mammary carcinomas may skew monocytes into a pro-tumorigenic phenotype to support tumor growth. SEMA7A could prove to be valuable in establishing new research avenues toward unraveling important tumor-host immune interactions in breast cancer patients.

## Introduction

Semaphorins (SEMAs) comprise a large family of transmembrane and secreted proteins that have been described as axon guidance molecules during neuronal development (Koppel et al., 1997; Pasterkamp and Kolodkin, 2003; Kikutani et al., 2007). Semaphorins, grouped into eight classes, are characterized by the presence of a conserved large SEMA domain (~500 amino acids) at the N-terminal domain and differentiated by their C-terminus (Koppel et al., 1997). Of the 8 classes of semaphorins, classes 1 and 2 are mostly found in invertebrates while classes 3–7 are found in vertebrates and the viral (V) class encoded by viruses. Emerging evidence is revealing additional roles for semaphorins in the immune system where they seem to exert diverse effects on leukocyte migration, adhesion, and inflammatory responses (Kikutani et al., 2007; Sakurai et al., 2010).

A growing body of evidence demonstrates the participation of classical neuronal developmental molecules in either tumor growth or inhibition by their effects on angiogenesis (Banu et al., 2006; Basile et al., 2006; Guttmann-Raviv et al., 2007; Acevedo et al., 2008; Sierra et al., 2008; Casazza et al., 2011). Semaphorins have been found to affect tumor progression by either modulating tumor angiogenesis, recruiting bone marrow cells that could then influence tumor progression, or by directly affecting the behavior of tumor cells. While some semaphorins were found to inhibit angiogenesis, others enhanced new blood vessel growth. Proangiogenic semaphorins include semaphorin 4A (SEMA4A), semaphorin 4D (SEMA4D), and semaphorin 5A (SEMA5A) (Capparuccia and Tamagnone, 2009). However, some members in semaphorin 3 (SEMA3) class have antiangiogenic effects (Basile et al., 2004; Varshavsky et al., 2008; Sadanandam et al., 2010; Sakurai et al., 2010; Meda et al., 2012). Although many classes of semaphorins have been studied in different cancers, the role of sempahorin7A (SEMA7A) in cancer progression is largely unknown. SEMA7A is a novel transmembrane GPI-anchored protein that has been described to function through plexin C1 and beta-integrins in multiple systems (Zhou et al., 2008). Recently, SEMA7A has been reported to be one of the proteins secreted by glioblastoma tumor cells that contribute to the highly invasive phenotype (Formolo et al., 2011). In this study we explore the role of SEMA7A in breast cancer progression using the DA-3 mammary tumor model. Specifically, we are investigating how SEMA7A can affect macrophage production of angiogenic molecules.

There is scarce information in literature on how SEMA7A affects macrophage induced angiogenesis. An angiogenic role for SEMA7A has been recently described to mediate vascular growth by bFGF stimulated fibroblasts in an experimental model of corneal neovascularization (Ghanem et al., 2011). In this manuscript using peritoneal elicited macrophages, a rich source of peripheral macrophages, we describe that SEMA7A induces macrophages to produce angiogenic molecules such as CXCL2/MIP-2 and that silencing the SEMA7A gene results in decreased production of these growth promoting molecules.

## Materials and methods

### Mice and cell lines

Female BALB/c mice were used in all studies (Charles River Laboratories, 8–12 week-olds), and were housed and used according to the National Institutes of Health guidelines, under protocols approved by Florida Atlantic University Institutional Animal Care and Use Committee. In these studies, we used the DA-3 cell line which was derived from the D1-DMBA-3 mammary tumor syngeneic to BALB/c mice and were provided by Dr. Diana M. Lopez, University of Miami School of Medicine, Miami, FL (Sotomayor et al., 1991). EpH4 mammary cells, a normal mammary cell line, were provided by Dr. Jenifer Prosperi, Indiana University School of Medicine-South Bend, IN. Both DA-3 and EpH4 cells were grown in complete DMEM media (DMEM with 10% FBS). RAW 264.7 cells (American Type Culture Collection, Manassas, VA, USA) were grown and maintained in RPMI 1640 containing 5% FBS as described previously (Nishiyama et al., 2006, 2008). Female BALB/c mice were inoculated in the lower right ventral quadrant with 7.5 × 10^5^ mammary tumor cells of the following types: (1) DA-3 cells silenced for the SEMA7A gene, (2) DA-3 cells with scramble shRNA, or (3) wild-type DA-3 cells. Imaging studies and caliper measurements of the primary tumors were performed up to 3 weeks post-tumor cell implantation and discontinued after this time point since the tumors become necrotic and fall off after 3 weeks. Tissues from 5-week tumor bearers were used in most of the studies, unless specified, based on our previous studies that production of tumor-derived factors peak at this time point (Lopez et al., 1991). At 5 weeks, tumors are not observed in the lung, liver, and bone. The establishment of metastastic colonies at distant sites occur at 10–12 weeks if 500–750 × 10^3^ cells are inoculated. For determination of angiogenesis by AngioSense (PerkinElmer, Waltham, MA), mice were implanted with SEMA7A shRNA silenced mammary tumor cells or scramble shRNA control mammary tumor cells and imaged at 21 days post-tumor implantation while tissues were collected at 5 weeks post-tumor cell implantation.

### Cell cultures

To obtain peritoneal elicited macrophages (PEMs), mice were injected intraperitoneally with 1.5 mL of 3% thioglycollate and 4 days post-thioglycollate injection and the peritoneal exudate cells were collected by peritoneal lavage with ice-cold RPMI 1640 with 10% fetal bovine serum. It is well-established that the optimal time point for harvesting PEMs is 4 days post-thioglycollate injection (Zhang et al., 2008). As our previous studies have shown increased chemokine and MMP-9 expression at 4–5 weeks post tumor cell inoculation, we chose 5 week time point to assess the role of SEMA7A in inducing proangiogenic factors by macrophages (Owen et al., 2003, 2011). PEMs from normal (N-PEM) and DA-3 tumor-bearing (DA-3 PEM) mice were then purified using CD11b magnetic beads (Miltenyi Biotec Inc., Auburn, CA). 2 × 10^6^ cells/mL were preconditioned by culturing with rmSEMA7A (5 μg/mL) (R&D Systems, Minneapolis, MN) and incubated for 24 h followed by stimulation with LPS (500 ng/mL) (Sigma Aldrich, St.Louis, MO) for an additional 12 h for RNA and 18 h for protein collection. RAW 264.7 macrophages were also conditioned as described above. For cell signaling inhibition studies, RAW 264.7 cells were pretreated with 1 μM MAPK inhibitor, U0126 (Calbiochem, inhibitors, EMD Millipore, Billerica, MA) for 1 h, conditioned with rmSEMA7A for 12 h and then stimulated with LPS (500 ng/mL) for an additional 12 h.

### Immunofluorescence

To determine the expression of SEMA7A, DA-3 mammary tumor cells were plated onto a confocal cover slide, post-fixed in 4% paraformaldehyde, blocked in 4% BSA and labeled with 0.1 μg/mL rat anti-SEMA7A (R&D Systems) followed by incubation in secondary antibody using donkey anti-rat IgG conjugated to AlexaFluor 488 (Molecular Probes, Eugene, OR). To visualize nuclei, DAPI (Vector Laboratories, Burlingame, CA) was added, cover-slipped with Vectashield and examined by confocal microscopy (Carl Zeiss Microimaging, Inc., Thornwood, NY).

### RNA isolation and real-time reverse transcriptase-polymerase chain reaction

Total RNA was extracted from murine tumor cells, RAW 264.7 macrophages or peritoneal elicited macrophages using the RNeasy Protect Mini Kit (QIAGEN) according to manufacturer's instructions. Briefly, cDNA was synthesized using Quantitech Reverse Transcription Kit (Qiagen, Valencia, CA) and gene expression was detected by SYBR Green real-time PCR analysis using SYBR RT^2^qPCR primers (Qiagen, proprietary primers, sequence not disclosed). The mRNA levels of gene of interest were normalized to β-actin mRNA levels. PCR cycles followed the sequence: 10 min at 95°C of initial denaturation; 15 s at 95°C; and 40 cycles of 1 min each at 60°C for annealing. The samples were amplified using the Strategene MX3005P cycler.

### Flow cytometry studies

The expression of CD11b and CD29 on macrophages was assessed by flow cytometry (FACS-Calibur, BD Biosciences, San Jose, CA). N-PEMs and DA-3 PEMs were stained by incubating with FITC-CD29 (0.125 μg/10^6^ cells) and APC-CD11b (0.1 μg /10^6^ cells) (both from BioLegend, San Diego, CA) for 20 min at 4°C. Surface expression was assessed by counting 10,000 cells and analyzed by FloJo software (Tree Star, Inc., Ashland, OR).

### Silencing of SEMA7A in macrophages

SEMA7A gene silencing in DA-3 PEMs was achieved by RNA interference via short hairpin RNA (Origene, Rockville, MD) as described above. Briefly, PT-67 packaging cells were transfected with one of the following plasmids: (1) plasmid encoding for shRNA sequence specifically for the SEMA7A gene and (2) scramble shRNA plasmid not specific for the SEMA7A gene, using Lipofectamine 2000 according to manufacturer's protocol. 0.45 μm filtered PT-67 transfected supernatants containing the retrovirus were used to silence SEMA7A gene in DA-3 PEMs for 36 h. Macrophages were then stimulated with LPS (100 ng/ml) for 12 h and q-PCR was performed to confirm SEMA7A gene silencing.

### Silencing of SEMA7A in DA-3 murine mammary tumor cells

Semaphorin 7A gene silencing in DA-3 mammary tumor cells was achieved using RNA interference via short hairpin RNA (Origene). A retrovirus shRNA plasmid system was used for stable SEMA7A gene knockdown. To generate the retrovirus infecting particles, PT-67 packaging cells were transfected with one of the following plasmids: (1) plasmid encoding for shRNA sequence specifically for the SEMA7A gene and (2) scramble shRNA plasmid not specific to the SEMA7A gene. Transfection was performed using standard Lipofectamine 2000 according to manufacturer's protocol. The different variants of transfected PT-67 cells were selected for 2 weeks with puromycin (2 μg/mL) and the cell-free/retrovirus-rich supernatants from the different PT-67 variants and controls were used to infect DA-3 cells for 24–48 h. The different DA-3 variants were then selected with puromycin (1 μg/mL) for 4 weeks. To confirm gene knockdown, real time quantitative polymerase chain reaction (q-PCR) (Qiagen) was performed using the SEMA7A specific primers according to manufacturer's protocol. Cells were passaged and selected until at least a 5-fold decrease in the SEMA7A gene expression was achieved when compared to the scramble control. The results of gene expression were then confirmed by western blotting for the SEMA7A protein.

### Monocyte migration assay

To test migration, RAW 264.7 murine monocytes were labeled with Calcein-AM (10 μM) and used in a modified Boyden Chamber assay. Briefly, 10^5^ RAW264.7 were placed in the transwell insert (8 μM pores) (BD Biosciences) of the upper chamber with lower chamber containing supernatants from: (1) DA-3 cells silenced for the SEMA7A gene, (2) DA-3 cells with scramble shRNA, and (3) wild-type DA-3 cells and incubated at 37°C in a CO_2_ incubator for 12 h. RAW 264.7 macrophage migration was measured using a plate reader set at an excitation wavelength of ~485 nm and an emission wavelength of ~520 nm. Absorbance values among the various groups were measured at least 2 times in triplicate and fitted to a 7-point standard curve.

### Protein determination

DA-3 murine mammary tumor cells were cultured under optimal conditions using DMEM culture media with 10% FBS until ~80% confluency was achieved. DA-3 tumor cells and DA-3 SEMA7A-silenced cells or intraperitoneal macrophages from 5-week DA-3 mammary tumor-bearing mice were lysed with sample buffer (20 mM dithiothreitol, 6% SDS, 0.25 M Tris, pH 6.8, 10% glycerol, 10 mM NaF and bromophenyl blue) and used to extract total protein. 20 μg of total protein from DA-3 cells and PEMs were resolved on 4–20% Mini-Protean SDS-PAGE gradient gels (BioRad Life Sciences, Hercules, CA) and transferred to PVDF membrane (Pierce, Rockford, IL) using a semi-dry transfer transblotter (BioRad) at 20 Volts for 40 min. The membrane was blocked overnight at 4°C in SeaBlock (Calbiochem), and subsequently incubated at room temperature with anti-mouse SEMA7A monoclonal antibody (1 μg/ml) (R&D Systems) and anti-mouse beta actin polyclonal antibody (0.25 μg/ml) (Li-Cor Biosciences, Lincoln, NE). Western blots were washed for 10 min three times with 0.5% Tween-PBS followed by 1 h incubation at room temperature with corresponding fluorescent antibodies (Li-Cor Biosciences). Blots were washed again for 10 min three times with 0.5% Tween-PBS and then dried at 37°C for 20 min. The membranes were then were visualized with Li-Cor imager. Protein concentration was normalized to beta-actin as loading control.

ELISAs of CXCL1, CXCL2/MIP-2 and MMP-9 (R&D Systems) were performed following manufacturer suggested protocol from DA-3 tumor control mice.

### Immunohistochemistry

Formalin-fixed tissue from controls, SEMA7A scramble controls and SEMA7A silenced tumors was paraffin embedded and sectioned at 4-micron thickness. Pre-treatment of formalin-fixed, paraffin-embedded tissue sections with heat-induced epitope retrieval (HIER) was done using diluted EnVision™ FLEX Target Retrieval Solution, High pH (50×) (Dako Omnis, Carpinteria, CA) following manufacturer protocol. The sections were deparaffinized and stained with hematoxylin and eosin (H&E) with automated Tissue Tek® 2000 processor (Sakura-Finetek, Torrance, CA). Adjacent tumor sections were assessed for vascularity using CD31 antibody. Dako FLEX monoclonal mouse anti-human CD31 antibody (diluted 1:30, DAKO) was used to highlight the vasculature of the tumors. CD31, expressed almost exclusively on endothelial cells, is a brown antibody stain against a hematoxylin counter stain. Photographs were taken at 50× magnification with mineral oil immersion using Olympus MDOB3 microscope and photographed with OlympusDP21 digital camera (Center Valley, PA).

### Tumor measurements and *in vivo* imaging for angiogenesis

Tumor size determination was performed by measuring the two longest perpendicular axes in the x/y plane of the tumor nearest to 0.1 mm by caliper measurement. The depth was assumed to be equivalent to the shortest of the perpendicular axes, defined as y and tumor volume = x(y)^2^/2. To account for vascularization in mice injected with either wild type DA-3 tumor cells or those silenced for SEMA7A, near infrared blood pool agent AngioSense 680 probe (2 nmol/mouse in 150 μL volume) (Perkin Elmer, Waltham, MA) was injected via tail vein 24 h before imaging. Mice were imaged using a bioluminescence optical imager (IVIS Lumina LTE, Perkin Elmer). Maximal near infrared signals were quantified using Living Image 2.5 (Xenogen, Perkin Elmer) image analysis software. Infrared signals are reported as photons/s.

#### Statistical analysis

Results are expressed as means ± standard deviation. Statistical analyses were performed using GraphPad Prism 3 software (LaJolla, CA). Statistical comparisons of paired groups were determined by Student's *t*-tests. Values of *p* < 0.05 were considered statistically significant.

## Results

### SEMA7A is expressed in DA-3 mammary tumor cells and expression is increased in peritoneal elicited macrophages of DA-3 mammary tumor-bearing mice

**Figure 1:**
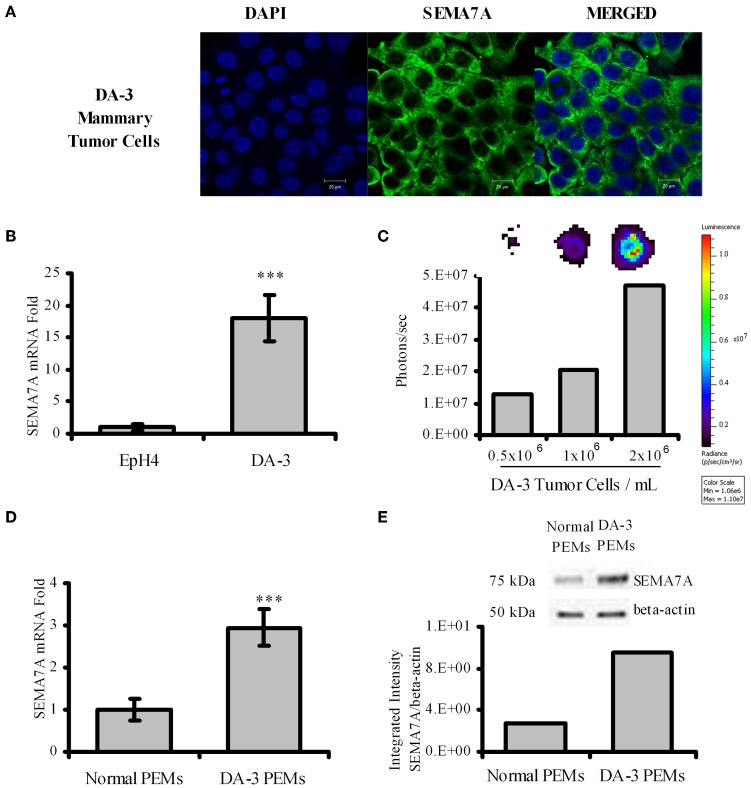
**DA-3 mammary tumor cells express SEMA7A**. **(A)** Shown is the confocal images of DA-3 mammary tumor cells for SEMA7A expression; **(B)** SEMA7A expression in EpH4 and DA-3 mammary tumor cells as determined by qPCR; **(C)** DOT blot analysis of DA-3 mammary tumor cell culture showing that SEMA7A is solubilized; **(D)** mRNA expression of SEMA7A in peritoneal elicited macrophages (PEMs) from normal and DA-3 mammary tumor-bearing mice; **(E)** Western blot analysis of total protein from PEMs from control and DA-3 mammary tumor bearers with 7 mice/group; also shown is integrated intensity graph of western blot indicating higher levels of SEMA7A expression in tumor bearers' PEMs. *In-vitro* experiments are representative of three independent experiments, ^***^p ≤ 0.001.

Semaphorins have been described to be expressed by various cell types. Although it is known that SEMA7A is expressed by monocytes, activated T cells, and keratinocytes, it is not known if tumor cells express SEMA7A. We therefore cultured DA-3 mammary tumor cells and assessed for SEMA7A expression. Confocal image shows that SEMA7A is expressed by the DA-3 mammary tumor cell line (Figure 1A). We then asked if SEMA7A is expressed by EpH4 mammary cells, a normal mammary cell line, and how do these levels compare with those in DA-3 tumor cells? qPCR revealed very low levels of SEMA7A expression in EpH4 cells compared to DA-3 mammary tumor cells (Figure 1B).

Members of the semaphorin family have been reported to be cleaved to generate soluble forms that have effects on immune function (Kumanogoh and Kikutani, 2003). It was not known if SEMA7A is solubilized in our tumor model. Since there are no reliable ELISAs available to quantify secreted SEMA7A protein, dot blot analysis was used to determine if SEMA7A is solubilized. Analysis of total protein from supernatants of 3 day DA-3 mammary tumor cell cultures confirmed the soluble protein expression of SEMA7A, with increased levels reflected by increased cell numbers (Figure 1C). It is possible that circulating levels of cleaved SEMA7A could have effects on other cells.

In the immune system, SEMA7A has been reported to be expressed in the myeloid and the lymphoid lineage cells (Delorme et al., 2005). There are no studies to date describing the expression of SEMA7A in macrophages of mammary tumor bearers. Thus, thioglycollate peritoneal elicited macrophages from normal (N-PEMs) and DA-3 mammary tumor-bearing mice (DA-3 PEMs) were therefore tested to determine SEMA7A expression. It is well-established that the optimal time point for peritoneal elicited macrophages is 4 days post-thioglycollate injection (Zhang et al., 2008). At earlier time points (e.g., 4–24 h post-thioglycollate) the majority of cells in the peritoneal cavity consist of neutrophils (Melnicoff et al., 1989; Lam et al., 2013). SEMA7A expression was determined at 3, 4, and 5 days post-thioglycollate injection in normal and DA-3 mammary tumor-bearing mice. There were no significant differences in SEMA7A expression at these days in peritoneal elicited cells from either normal or tumor-bearing mice. We therefore opted for 4 days as our set time point for these studies. A 3-fold increase in SEMA7A expression at the mRNA level was found in peritoneal elicited macrophages from DA-3 PEMs (Figure 1D) compared to the expression in N-PEMs. Similarly, increased protein expression of SEMA7A was found in DA-3 PEMs compared to normal PEMs (Figure 1E). Quantification of the bands from western blot analysis confirmed increased SEMA7A protein expression in DA-3 PEMs.

### Expression of SEMA7A receptor, β1 integrin (CD29) is increased in DA-3 mammary tumor cells and macrophages from mammary tumor-bearing mice

**Figure 2:**
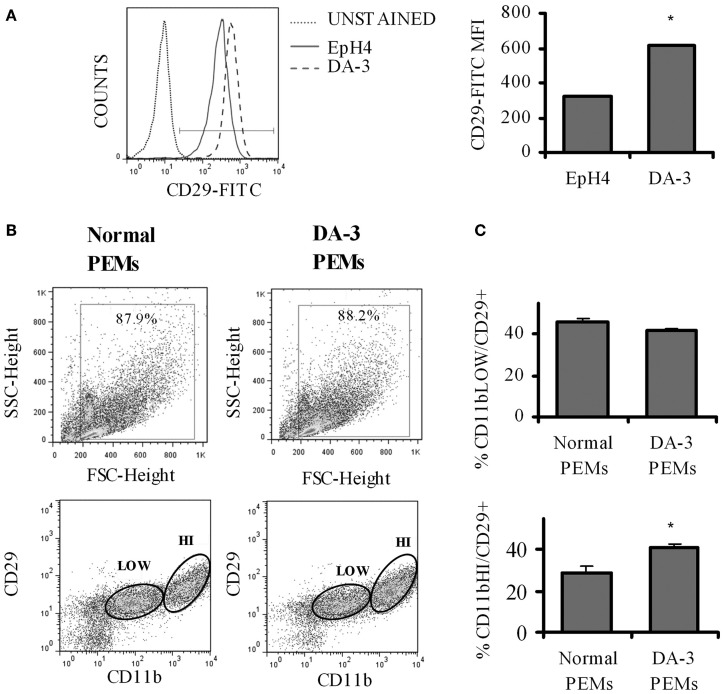
**β1 integrin expression is increased in DA-3 cells and macrophages of mammary tumor-bearing mice. (A)** Flow cytometric analysis of DA-3 mammary tumor cells and EpH4 mammary epithelial cells for the expression of β1 integrin (CD29) (a representative plot of 2 independent experiments); **(B)** scatter plot of CD11b^+^ and CD29 expression in PEMs from normal and DA-3 mammary tumor-bearing mice; **(C)** flow cytometric analysis of PEMs from control and DA-3 mammary tumor bearers gated on either CD11b^low^ or CD11b^high^ and assessed for the expression of CD29, *N* = 8, ^*^p ≤ 0.05.

The principal signaling function of SEMA7A in the nervous and immune systems is mediated through α1β 1 integrin (Pasterkamp et al., 2003; Suzuki et al., 2007; Gan et al., 2011). Increased β 1 signaling has previously been shown to be associated with decreased survival in invasive breast cancer (Yao et al., 2007). We first determined if there is a differential β 1 integrin expression in EpH4 and DA-3 mammary tumor cells. Flow cytometric analysis showed that even though the percentage of β 1 integrin (CD29) positive cells remained unchanged between the normal EpH4 cells and the DA-3 mammary tumor cells, the mean fluorescence intensity was almost doubled in the tumor cells (Figure 2A). The expression of SEMA7A's receptor, β 1 integrin, in peripheral macrophages between normal and tumor bearers has not yet been well characterized. We determined if there are altered levels of β 1 integrin expression in peritoneal elicited macrophages (PEMs) from normal and DA-3 mammary tumor-bearing mice. PEMs were gated based on the fluorescent intensity of CD11b expression (Figure 2B). Flow cytometric analysis of CD11b^low^ PEMs from normal and DA-3 tumor bearing mice revealed no significant differences in the frequency of CD29^+^ cells (Figure 2C). In contrast, expression of CD11b^hi^CD29^+^ in DA-3 PEMs was higher (*p* < 0.05) compared to the expression in normal PEMs (Figure 2C).

### Treatment of macrophages with rmSEMA7A induces production of angiogenic CXCL2/MIP-2

**Figure 3:**
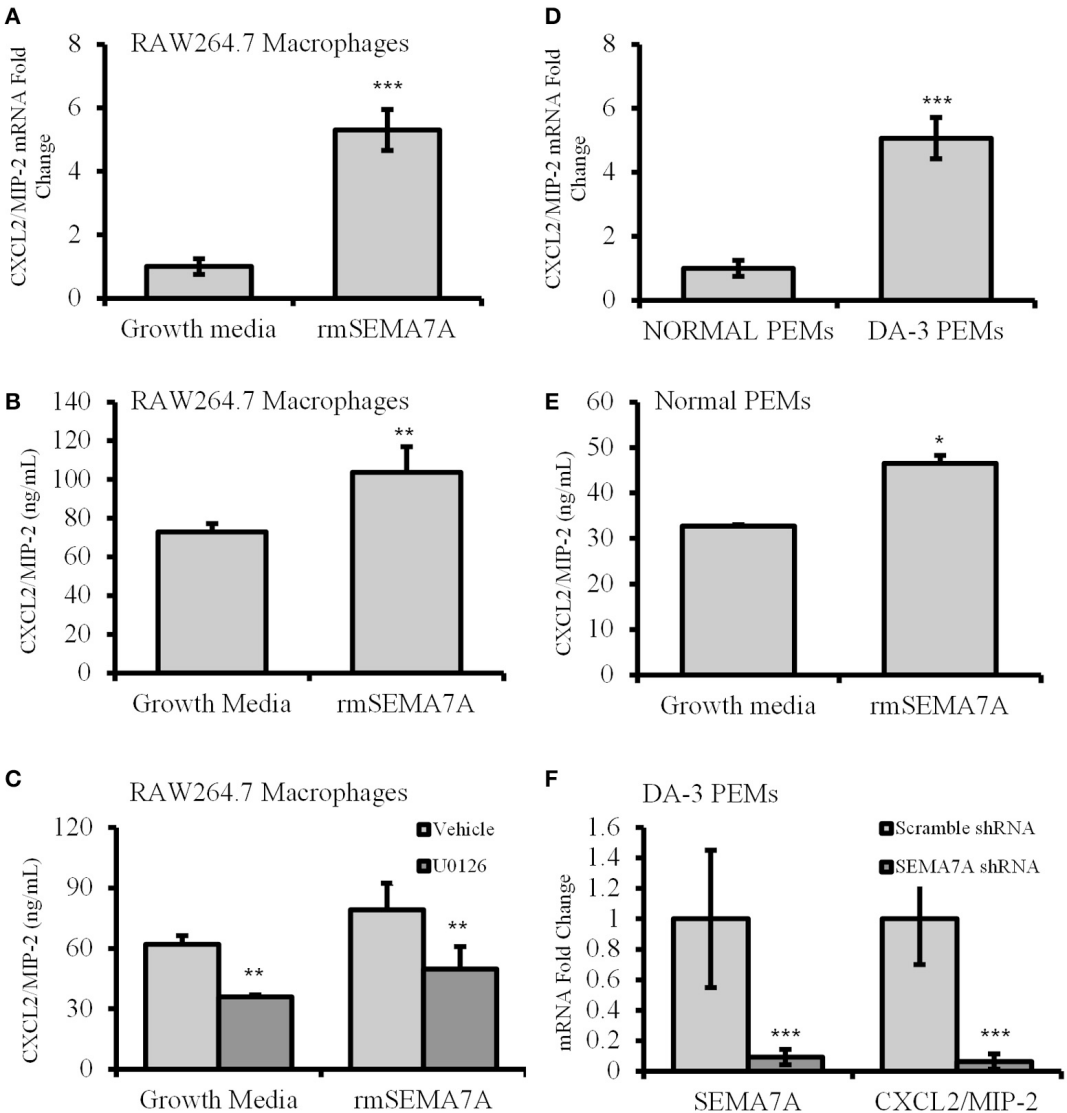
**Increased expression of angiogenic chemokine, CXCL2/MIP-2 in rmSEMA7A treated RAW264.7 macrophages and peritoneal elicited macrophages from mammary tumor-bearing mice**. The effect of treatment with rmSEMA7A in RAW264.7 macrophages is shown in panels **(A–C)**. Expression of CXCL2/MIP-2 at mRNA level **(A)** and at protein level determined by ELISA **(B)** is increased in RAW 264.7 macrophages treated with rmSEMA7A; and **(C)** effect of treatment with U0126, a MAPK inhibitor (1 μM) on CXCL2/MIP-2 expression by RAW 264.7 macrophages treated with rmSEMA7A. CXCL2/MIP-2 expression is increased in peritoneal elicited macrophages (PEMs) from DA-3 mammary tumor bearers compared to N-PEMs: **(D)** increased mRNA expression of CXCL2/MIP-2 in DA-3 PEMs compared to PEMs from normal as determined by qPCR, *N* = 8; **(E)** CXCL2/MIP-2 ELISA of normal PEMs treated with rmSEMA7A and stimulated with LPS; and **(F)** the effect of SEMA7A gene knockdown in DA-3 PEMs on mRNA expression of SEMA7A and CXCL2/MIP-2 as determined by qPCR. *In-vitro* experiments are representative of three independent experiments. ^*^p ≤ 0.05, ^**^p ≤ 0.01, ^***^p ≤ 0.001.

Macrophages from tumor-bearing mice are known to produce angiogenic molecules (Mantovani et al., 1992). Previous studies have shown that tumor-derived factors induce macrophages to produce angiogenic and proinflammatory molecules (Pollard, 2004). Holmes et al. have shown that SEMA7A induces the production of proinflammatory molecules including the IL-8 homolog of chemokine CXCL2/MIP-2, which also has angiogenic properties (Holmes et al., 2002). As shown in the previous section, DA-3 mammary tumor cells express and shed SEMA7A. We therefore determined whether soluble SEMA7A has an effect on macrophage function. Toward these studies we used the macrophage cell line RAW 264.7 in which SEMA7A mRNA was undetectable (CT value > 37). RAW264.7 macrophages, as a model of tissue macrophages isolated from normal mice, have been used frequently for *in vitro* studies of macrophage function. qPCR analysis of RAW 264.7 macrophages preconditioned with rmSEMA7A revealed that expression of proangiogenic molecules CXCL2/MIP-2 was increased by 5-fold (*p* < 0.001) (Figure 3A) after LPS stimulation. We found a significant (*p* < 0.01) increase in CXCL2/MIP-2 protein in RAW 264.7 macrophages treated with rmSEMA7A and LPS (Figure 3B). These studies also included culturing of RAW 264.7 cells with rmSEMA7A alone, which also showed an increase in CXCL2/MIP-2 (data not shown). SEMA7A has previously been reported to function through β 1 integrin activation of MAPK signaling pathway to promote monocyte inflammatory response (Suzuki et al., 2007). To get insight if SEMA7A induces CXCL2/MIP-2 via MAPK pathway, RAW 264.7 macrophages were pretreated with a MAPK inhibitor (U0126). We found that U0126 conditioned and rmSEMA7A treated cells exhibited decreased (*p* < 0.01) production of CXCL2/MIP-2 compared to those cultured with rmSEMA7A alone (Figure 3C).

To determine if freshly isolated macrophages from normal and DA-3 mammary tumor bearers express CXCL2/MIP-2, peritoneal elicited macrophages from normal and DA-3 mammary tumor bearers were obtained and assessed for CXCL2/MIP-2 expression by qPCR. A greater than 5-fold increase (*p* < 0.001) in CXCL2/MIP-2 expression was observed in DA-3 PEMs compared to normal PEMs (Figure 3D). We have previously shown that tumor-derived factors have an effect on profile of PEMs (Lopez et al., 1996; DiNapoli et al., 1997; Handel-Fernandez et al., 1997; Torroella-Kouri et al., 2003). Therefore, peritoneal elicited macrophages were used as we wanted to determine the effect of SEMA7A in circulation on macrophages. Since RAW 264.7 macrophages treated with rmSEMA7A had increased expression of CXCL2/MIP-2, we determined if treatment of N-PEMs with rmSEMA7A had an effect on production of angiogenic molecule, CXCL2/MIP-2. A considerably (*p* < 0.05) enhanced expression of CXCL2/MIP-2 was observed in N-PEMs pretreated with rmSEMA7A and then stimulated with LPS (Figure 3E). Given that SEMA7A is known to induce CXCL2/MIP-2, and PEMs from DA-3 mammary tumor bearers have increased CXCL2/MIP-2 and SEMA7A, we silenced the SEMA7A gene in DA-3 PEMs using shRNA. Effectiveness of SEMA7A gene silencing as indicated in the 1st set of bars shows that SEMA7A gene was significantly (*p* < 0.001) silenced compared to the scramble control (Figure 3F). SEMA7A gene silenced DA-3 PEMs expressed significantly less CXCL2/MIP-2 compared to scramble control as determined by q-PCR (Figure 3F). It is important to note that our previous studies show that DA-3 cells express CXCL2/MIP-2. It is possible that SEMA7A could function in an autocrine manner to upregulate the expression of CXCL2/MIP-2.

### Decreased tumor-derived SEMA7A results in reduced *in vitro* macrophage migration and CXCL2/MIP-2 production

**Figure 4:**
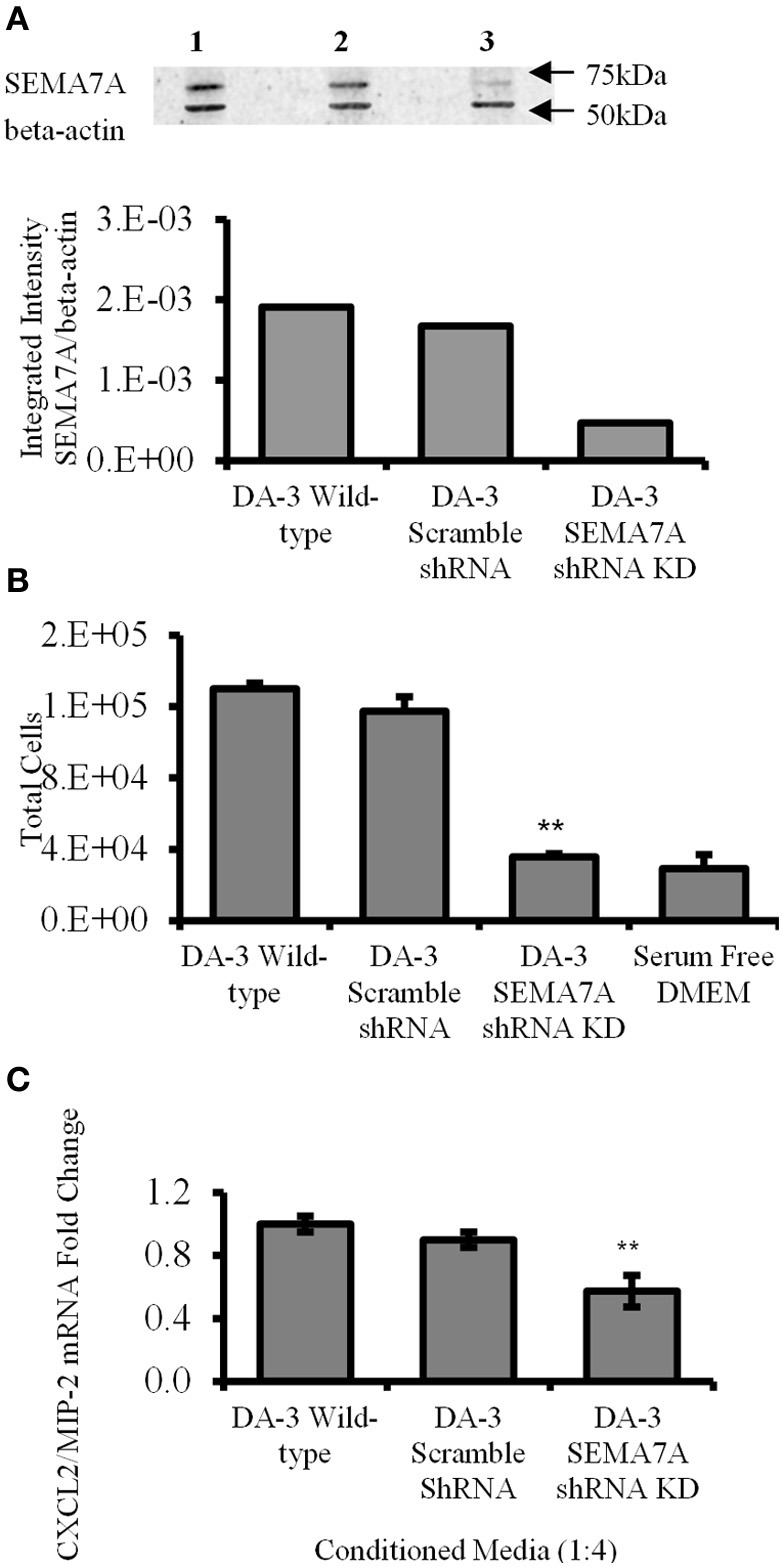
**Decreased tumor-derived SEMA7A results in reduced *in vitro* macrophage migration and CXCL2/MIP-2 production**. **(A)** Effect of SEMA7A gene silencing on SEMA7A expression in DA-3 mammary tumor cells as determined by western blot analysis; **(B)** migration of Calcein AM labeled RAW 264.7 macrophages toward cell-free supernatants from either wild type DA-3 tumor cells, SEMA7A scramble shRNA DA-3 tumor cells, SEMA7A shRNA knockdown DA-3 cells or serum free media alone. **(C)** mRNA expression of CXCL2/MIP-2 on macrophages from normal mice *in vitro* treated with conditioned media from either wild type, SEMA7A scramble or SEMA7A knockdown DA-3 mammary tumor cells. *In-vitro* experiments are representative of three independent experiments. ^**^p ≤ 0.01.

Holmes et al. demonstrated that SEMA7A is a potent monocyte chemoattractant with 1000-times greater chemotactic activity than monocyte chemotactic protein, MCP-1. (Holmes et al., 2002). We hypothesized that silencing SEMA7A gene in DA-3 mammary tumor cells would result in decreased secretion of SEMA7A in tumor cell cultures and treatment of macrophages with this conditioned media would therefore have a negative influence on their migration. Thus, SEMA7A gene was silenced in DA-3 mammary tumor cells by shRNA. Western blotting was performed to test the effectiveness of SEMA7A gene silencing. Lane 1 indicates DA-3 wild type, lane 2 shows DA-3 scramble shRNA and lane 3 consists of DA-3 SEMA7A shRNA knockdown (Figure 4A, top panel). Integrated intensity graphs show a 6-fold decrease in SEMA7A expression in DA-3 SEMA7A shRNA knockdown cells compared to either DA-3 wild type or DA-3 scramble shRNA cells (Figure 4A, bottom panel). Although DA-3 cells express lower levels of CXCL2/MIP-2 compared to macrophages, silencing the SEMA7A gene also lead to a decrease in tumor-derived CXCL2/MIP-2. To determine if SEMA7A plays a role in monocyte migration, a modified Boyden chamber assay was performed using RAW 264.7 murine macrophages and conditioned media from wild type DA-3 tumor, DA-3 scramble shRNA, or DA-3 SEMA7A shRNA knockdown cells as possible chemoattractants. Fewer number of RAW 264.7 monocytes migrated towards the conditioned media from SEMA7A silenced DA-3 cells compared to media from either wild type DA-3 tumor cells or DA-3 cells with scramble shRNA (Figure 4B). Since we demonstrated that DA-3 mammary tumor cells produce SEMA7A, and that treatment of macrophages with rmSEMA7A induced the production of proangiogenic CXCL2/MIP-2, we hypothesized that silencing SEMA7A gene in DA-3 mammary tumor cells would have an inhibitory effect on production of CXCL2/MIP-2 by macrophages treated with tumor cell supernatants silenced for the SEMA7A gene. We therefore tested to see if SEMA7A gene silencing in tumor cells has an effect on CXCL2/MIP-2 chemokine expression. In macrophage cultures with conditioned media from SEMA7A shRNA knockdown DA-3 cells, there was a significant (*p* < 0.01) reduction in CXCL2/MIP-2 expression compared to the cultures with SEMA7A (Figure 4C).

### Decreased tumor growth in mice bearing SEMA7A silenced mammary tumors

**Figure 5:**
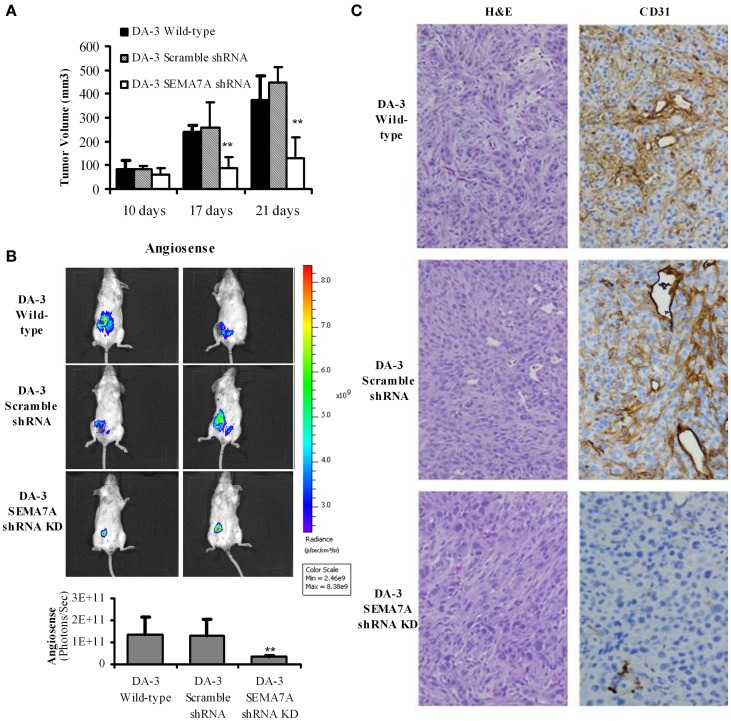
**Decreased tumor growth and angiogenesis in mice bearing SEMA7A silenced mammary tumors. (A)** Tumor volume is decreased in mice bearing SEMA7A shRNA knockdown DA-3 mammary tumor compared with either wild type or SEMA7A scramble shRNA DA-3 mammary tumors; **(B)** ventral image of DA-wild type, SEMA7A scramble shRNA DA-3 or SEMA7A shRNA knockdown DA-3 mammary tumor bearing mice showing 1 out 20 mice/group; quantification (photons/sec) of AngioSense specific fluorescent signal indicating decreased angiogenesis at 21 days post-tumor implantation in the SEMA7A knockdown group, *N* = 10 per group, repeated twice ^**^p ≤ 0.01. **(C)** H&E and CD31 staining in tumor sections from DA-3 wild type, scramble shRNA and SEMA7A shRNA knockdown tumor sections. Significance is indicated ^*^p ≥ 0.01.

Culturing of RAW 264.7 or thioglycollate elicited macrophages with rmSEMA7A induced the expression of CXCL2/MIP-2, a pro-angiogenic chemokine. We have previously shown that mice bearing either the parental D1-DMBA-3 or DA-3 mammary tumors exhibit higher levels of pro-angiogenic molecules (Owen et al., 2011). It is well-established that angiogenesis is required for invasive tumor growth and that tumors do not grow more than 1 mm^3^ in the absence of angiogenesis (Folkman, 1971). We have shown in the previous section that SEMA7A induces production of angiogenic molecules by macrophages. We therefore determined if implantation of BALB/c mice with SEMA7A knockdown DA-3 mammary tumors has an inhibitory effect on tumor growth. To determine the *in vivo* role of SEMA7A, mice were implanted with either wild-type DA-3, scramble shRNA DA-3, or SEMA7A gene knockdown DA-3 (SEMA7A KD) mammary tumor cells. Mice implanted with SEMA7A KD tumors had significantly (*p* < 0.01) decreased primary tumor volume compared to the wild type or SEMA7A scramble control DA-3 mammary tumors (Figure 5A). Since SEMA7A KD tumors had lower tumor volume, we tested to see if there is decreased angiogenesis in these mice by use of AngioSense fluorescent probe and CD31 staining by immunohistochemistry. Thus, an AngioSense fluorescent probe was used to determine the extent of angiogenesis in the tumors by an *in vivo* imaging system. Shown in the upper panel are mice bearing wild type DA-3 tumors; the middle panel, scramble control for shRNA; while the bottom panel shows mice bearing SEMA7A KD tumors. Significantly (*p* < 0.01) decreased angiogenesis was observed in mice bearing the SEMA7A KD tumors compared to the scramble controls or wild type DA-3 mammary tumors (Figure 5B). We also show the quantification results of *in vivo* imaging indicating a similar trend in tumor growth. Decrease in angiogenesis in SEMA7A KD tumor sections was also observed by immunohistochemistry. H&E and immunohistochemical staining for CD31 highlighted angiogenesis in control tumors but minimally in SEMA7A KD tumors (Figure 5C).

### Peritoneal elicited macrophages from mice bearing SEMA7A KD tumors produce decreased levels of angiogenic molecules

**Figure 6:**
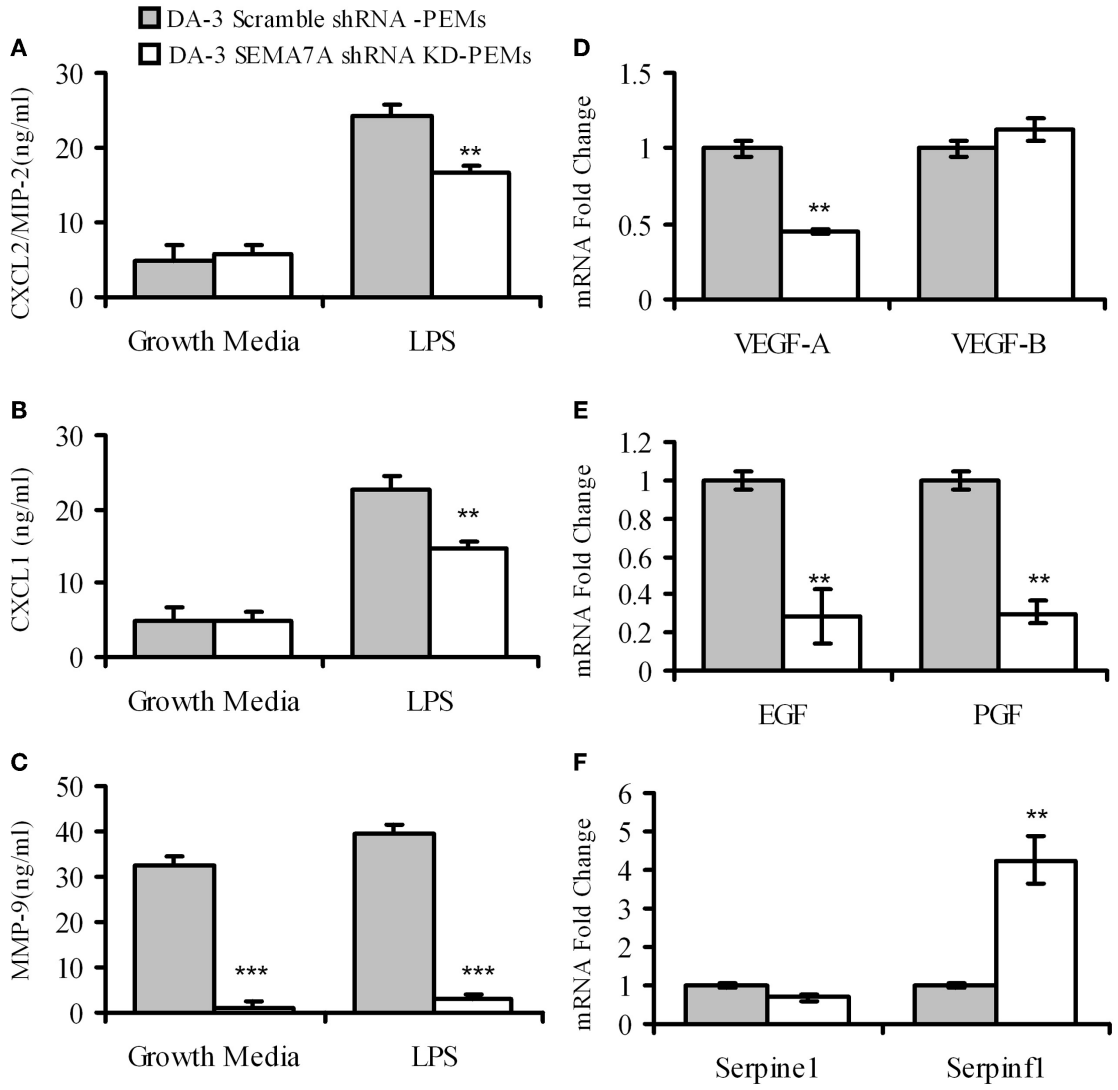
**Macrophages from mice bearing SEMA7A shRNA knockdown tumors produce decreased levels of angiogenic molecules**. ELISA of peritoneal macrophages from scramble shRNA DA-3 and SEMA7A shRNA knockdown DA-3 tumor bearers cultured with and without LPS for protein levels of: **(A)** CXCL2/MIP-2; **(B)** CXCL1; and **(C)** MMP-9, *N* = 16. qPCR of peritoneal macrophages from scramble shRNA DA-3 and SEMA7A shRNA knockdown DA-3 tumor bearers for mRNA expression of: **(D)** VEGFA and VEGFB; **(E)** EGF and PGF; and **(F)** Serpine 1 and Serpinfl, *N* = 6, repeated twice. ^**^p ≤ 0.01, ^***^p ≤ 0.001.

4–5 weeks post-tumor cell implantation, thioglycollate elicited macrophages from DA-3 scramble shRNA control or DA-3 SEMA7A shRNA mammary tumor-bearing mice were analyzed for the production of pro-angiogenic chemokines CXCL2/MIP-2, CXCL1 and matrix metalloprotease MMP-9. LPS-stimulated macrophages from mice implanted with SEMA7A gene silenced DA-3 mammary tumors produce significantly (*p* < 0.01) lower amounts of pro-angiogenic molecules compared to those implanted with SEMA7A scramble control DA-3 tumor cells. While there were no major differences in secretion of CXCL2/MIP-2 and CXCL1 in unstimulated macrophages from either SEMA7A scramble control or SEMA7A silenced mammary tumor-bearing mice, there were significant (*p* < 0.01) differences in the production of both these chemokines from LPS-stimulated (100 ng/ml) cultures (Figures 6A,B). Thus, LPS stimulated macrophages from scramble control DA-3 mammary tumors produced ~25 ng/mL of CXCL2/MIP-2 while those from SEMA7A silenced tumor bearers produced ~18 ng/mL (Figure 6A). Similarly, LPS stimulated macrophages from scramble controls produced ~24 ng/mL and those from SEMA7A silenced DA-3 tumor-bearers' macrophages produced 16.8 ng/mL of CXCL1 (Figure 6B). Interestingly, implantation of SEMA7A knockdown tumor cells decreased the production of MMP-9 by intraperitoneal macrophages in both unstimulated and LPS-stimulated cultures (Figure 6C). Furthermore, we assayed a series of tumorigenesis-related genes by qPCR on peritoneal macrophages from SEMA7A shRNA KD or shRNA scramble control DA-3 mammary tumor-bearing mice. PEMs from SEMA7A KD tumor bearing mice showed a significant reduction in VEGF-A expression but not VEGF-B expression (Figure 6D). In contrast, expression of both epidermal growth factor (EGF) and platelet growth factor (PGF) was significantly reduced in PEMs from SEMA7A KD tumor bearing mice (Figure 6E). Interestingly, the levels of serpinf1, a secreted protein that has both anti-angiogenic and anti-tumorigenic functions, was significantly increased in PEMs from SEMA7A KD tumor bearers (Figure 6F).

## Discussion

The biological role of SEMA7A in breast cancer progression was explored in this study. First, we find that SEMA7A is expressed by mammary tumor cells. Second, we show that SEMA7A expression is upregulated in macrophages of mammary tumor-bearing mice. Third, we demonstrate that SEMA7A induces the expression of proangiogenic molecule CXCL2/MIP-2 in macrophages. Fourth, we find decreased tumor growth in mice implanted with SEMA7A shRNA DA-3 mammary tumor cells. Lastly, we find that there is decreased angiogenesis in mice implanted with SEMA7A knockdown mammary tumors. These findings suggest that SEMA7A could have a direct effect on tumor cell growth and macrophage function. We are the first to show that SEMA7A plays a role in breast cancer progression.

SEMA7A was first identified in the immune system, as myeloid and lymphoid lineage cells have been reported to express this molecule (Comeau et al., 1998; Lange et al., 1998; Xu et al., 1998). There are very few reports on SEMA7A expression as it relates to cancer. We are the first to clearly demonstrate that SEMA7A is expressed by mammary tumor cells. Formolo et al. identified SEMA7A as one of the proteins in highly invasive astrocytoma cell line U87 while the less aggressive cells do not express this protein (Formolo et al., 2011). Our results parallel with these results as DA-3 mammary tumor cells had greater intensity in expression of this SEMA7A compared to the nontumorigenic mammary EpH4 cells. This raises the possibility that metastatic tumors express higher levels of SEMA7A. We are actively pursuing this in our laboratory by assessing different breast tumor cell lines with varying levels of metastatic potential for SEMA7A expression and correlating with aggressive behavior. Interestingly, while SEMA7A is known to affect monocyte activation *in vitro* via β 1 integrin–mediated effects (Holmes et al., 2002), the role of SEMA7A in the activation of tumor cells has not yet been studied. We found that while PEMs from normal mice express low levels of SEMA7A, the expression of this protein is increased in PEMs from tumor bearers. So what induces the expression of this molecule in macrophages? In a murine fibrosis model, TGF-β has been reported to induce the expression of SEMA7A in the murine lung (Kang et al., 2007). We are testing tumor- and/or host-derived factors in inducing SEMA7A expression in PEMs.

Although the identification of SEMA7A receptors remains controversial, two potential receptors have been identified, i.e., plexin C1 and the β 1 subunit of integrin receptor. The biological activities of SEMA7A in the immune system have only recently been elucidated. SEMA7A induces the production of inflammatory cytokines such as IL-6, TNF-α and IL-8 (Suzuki et al., 2007), an effect that could be mediated through direct interaction of GPI-anchored SEMA7A protein with α1β 1 integrins on target cells. Alternatively, SEMA7A could be cleaved by ADAM-17 and have paracrine effects on other cells. Cell surface bound semaphorins have been found to be proteolytically cleaved in order to exert their biological function. For example, in order to exert proangiogenic effect, SEMA4D is proteolytically cleaved by membrane type 1-matrix metalloproteinase, and the resulting soluble form acts on endothelial cells to enhance angiogenesis (Henningsen et al., 2010). SEMA7A is a GPI-anchored protein that has been found to be cleaved in platelets by ADAM-17 (Fong et al., 2011). We have previously reported increased expression of ADAM-17 in mammary tumor-bearing mice (Owen et al., 2003). It is possible that ADAM-17 in the tumor bearers could affect cleavage of SEMA7A. Biological effects of SEMA7A have been reported to function through both the soluble and membrane forms. Soluble SEMA7A has been shown to be an extremely potent monocyte chemoattaractant (Holmes et al., 2002) while membrane bound SEMA7A has been reported to stimulate monocytes and macrophages through α1β 1 integrin and increase production of proinflammatory cytokines including IL-6 and TNF-α (Suzuki et al., 2007). SEMA7A has been shown to promote spreading and dendricity in human melanocytes through its receptor, β 1-integrin. In this study, we report that peritoneal elicited macrophages from mammary tumor-bearing mice express higher levels of β1 integrins as well as its ligand SEMA7A compared to the control mice in tumor bearers' macrophages, suggesting that SEMA7A could function in a paracrine manner. In a cancerous system, it is probable that SEMA7A could mediate its functions through both membrane and soluble forms.

We have previously shown that macrophages from mammary tumor-bearing mice produce angiogenic molecules in response to tumor-derived factors (Libreros et al., 2012). Angiogenesis plays a crucial role in growth of tumors since solid tumors cannot grow beyond 1–2 mm^3^ without establishing an adequate blood supply (Folkman, 1971). Using immunohistochemistry and an AngioSense probe, an *in vivo* blood pool vascular fluorescent imaging agent, we determined the *in vivo* role of SEMA7A by comparing angiogenesis in mice bearing scramble shRNA DA-3 mammary tumors with those bearing SEMA7A shRNA knockdown DA-3 mammary tumors. Since these studies showed a significant reduction in tumor volume in SEMA7A shRNA knockdown DA-3 mammary tumors, we hypothesized that these mice would produce decreased levels of angiogenic molecules. It is also possible that although we have knocked down the gene in the tumor cells, host derived SEMA7A may contribute toward angiogenesis. Using SEMA7A knockout mice, we are determining the effects of tumor-derived *vs*. host-derived SEMA7A.

Axonal guidance molecule expression is dysregulated in many types of cancer, including breast cancer, suggesting that they may be excellent targets for effective therapeutic strategies (Harburg and Hinck, 2011). In this report we provide novel data showing that macrophages from SEMA7A shRNA knockdown mammary tumor bearers have decreased production of angiogenic chemokines CXCL2/MIP-2 and CXCL1 as well as matrix degrading enzyme, MMP-9. Although it is known that cytokines such as TNF-α induce MMP-9 through MAPK pathway (Holvoet et al., 2003; Moon et al., 2004), there are no studies in literature describing induction of MMPs by SEMA7A. (Guo and Giancotti, 2004). We are the first to show a relationship between MMP-9 and SEMA7A. We speculate that SEMA7A-β 1 integrin ligation may activate MAPK pathway. Activation of MAPK pathway has been shown to play an important role in tumor invasion and metastasis via interaction of integrins with specific receptors (Guo and Giancotti, 2004). Further, integrins have been reported to associate with receptor tyrosine kinases (RTKs) to activate signaling pathways, including MAPK pathways that are necessary for tumor invasion and metastasis. We have also shown that macrophages from SEMA7A shRNA knockdown mammary tumor bearers have increased levels of serpinf1, a secreted protein known to have anti-angiogenic and anti-tumorigenic functions (Filleur et al., 2009). It is possible that SEMA7A could act in an autocrine manner to upregulate the expression of not only angiogenic molecules, but also the integrins to enhance metastatic growth. We are now characterizing the effect of SEMA7A on different mammary tumor cells and their ability to migrate and metastasize. These findings could lead to further studies in the role of Semaphorin 7A in tumor progression in breast and many other cancers.

### Conflict of interest statement

The authors declare that the research was conducted in the absence of any commercial or financial relationships that could be construed as a potential conflict of interest.
